# LandmarkNet: a 2D digital radiograph landmark estimator for registration

**DOI:** 10.1186/s12911-020-01164-4

**Published:** 2020-07-21

**Authors:** Zhen Wang, Cong Liu, Longhua Ma

**Affiliations:** 1 College of Control Science and Engineering, Zhejiang University, Yugu Road, Hangzhou, 310013 China; 2 NingboTech University, No.1 Qianhu South Road, Ningbo, 315100 China

**Keywords:** Keypoint estimation, Intermediate supervision, Heatmap

## Abstract

**Background:**

Radiation therapy requires precision to target and escalate the doses to affected regions while reducing the adjacent normal tissue exposed to high radiotherapy doses. Image guidance has become the start of the art in the treating process. Registering the digital radiographs megavoltage x ray (MV-DRs) and the kilovoltage digital reconstructed radiographs (KV-DRRs) is difficult because of the poor quality of MV-DRs. We simplify the problem by registering between landmarks instead of entire image information, thence we propose a model to estimate the landmark accurately.

**Methods:**

After doctors’ analysis, it is proved that it is effective to register through several physiological features such as spinous process, tracheal bifurcation, Louis angle. We propose the LandmarkNet, a novel keypoint estimation architecture, can automatically detect keypoints in blurred medical images. The method applies the idea of Feature Pyramid Network (FPN) twice to merge the cross-scale and cross-layer features for feature extraction and landmark estimation successively. Intermediate supervision is used at the end of the first FPN to ensure that the underlying parameters are updated normally. The network finally produces heatmap to display the approximate location of landmarks and we obtain accurate position estimation after non-maximum suppression (NMS) processing.

**Results:**

Our method could obtain accurate landmark estimation in the dataset provided by several cancer hospitals and labeled by ourselves. The standard percentage of correct keypoints (PCK) within 8 pixels of estimation for the spinous process, tracheal bifurcation and Louis angle is 81.24%, 98.95% and 85.61% respectively. For the above three landmarks, the mean deviation between the predicted location of each landmark and corresponding ground truth is 2.38, 0.98 and 2.64 pixels respectively.

**Conclusion:**

Landmark estimation based on LandmarkNet has high accuracy for different kinds of landmarks. Our model estimates the location of tracheal bifurcation especially accurately because of its obvious features. For the spinous process, our model performs well in quantity estimation as well as in position estimation. The wide application of our method assists doctors in image-guided radiotherapy (IGRT) and provides the possibility of precise treatment in the true sense.

## Background

During a radiation treatment guided by medical image, it is essential to positioning patients accurately by image registration which overlays two or more images of the same scene taken at different times, from different viewpoints, and/or by different sensors [1]. The effect of registration is directly related to the effect of treatment. In the process of image-guided radiotherapy, it is usually registered by MV-DRs (Digital Radiography, generated by mega-level X-rays through the human body on the Electronic Portal Imaging Device) and KV-DRRs (Digitally Reconstructed Radiography, re-projected from computed tomography of kilovolt X-ray), so that the treatment position is aligned with the planned position for precise radiotherapy. MV-DRs images are widely used because of the advantages of fast imaging, easy storage and convenient post-processing. In MV-DRs images, the contrast between the bone tissue and soft tissue is low, the bone contour is blurred, and the soft tissue is obvious. But the bone tissue in KV-DRRs images has high contrast and clear edges. The registration of the two images is very technically demanding for the physician because of the poor quality of the MV-DRs. In this paper, the landmarks in the image (spinous process, tracheal bifurcation, etc.) are extracted, and the registration of the two images is achieved by landmark alignment, which reduces the deviation caused by the different postures of the patient at different time periods, avoids the interference of the non-interest area on the attention area. Due to the disadvantages of unclear of MV-DRs, it is often necessary to process the original MV-DRs into a form that is easier to register, Liu and et al. [2] developed a fractal convolutional network to synthesizing KV-DRRs from MV-DRs for registration based on mutual information. This article is registered through key points. Estimation of key points has many applications in pulmonary nodules, The work by Shi et al. [3] input segmented lung image to conventional neural networks for extract the feature of pulmonary nodules and adopted position-sensitive score maps to represent the location information of lung nodules.

Key points detection is one of the basic algorithms of computer vision. It plays a fundamental role in the research of other related fields of computer vision, and is widely used in the fields of face alignment and pose estimation. There are three methods for detecting face key points, which are the traditional methods of Active Shape Model [4] and Active Appearance Model [5, 6]; methods based on cascading shape regression [7]; based on deep learning methods [8–13]. Pose estimation is usually also considered as a detection problem, and the output is heatmap [14]. The Stacked Hourglass Networks for pose estimation proposed by Newell et al. [15] outputs the precise pixel position of the human key points for a given single RGB image, and use multi-scale features to capture the spatial position information of each joint point of the human body. In the keypoint estimation subnet of MultiPoseNet, proposed by Kocabas et al. [16], takes hierarchical CNN features (outputted by the corresponding Feature Pyramid Networks [17]) and outputs keypoint and segmentation heatmaps. We also use the method of generating heatmaps to estimate landmarks. This work proposes a multi-scale deep CNN, denoted the LandmarkNet, for the detection of key points in medical images. Our network generates three sets of feature pyramids with two lateral connections to merge the cross-scale and cross-layer features. The network detects the landmarks on the feature map output by the feature pyramid and represent it with a heatmap. The accurate locations of landmark can be obtained by non-maximum suppression The result of our method will assist doctors in image-guided radiotherapy, especially during the registration phase.

## Methods

**Fig. 1:**
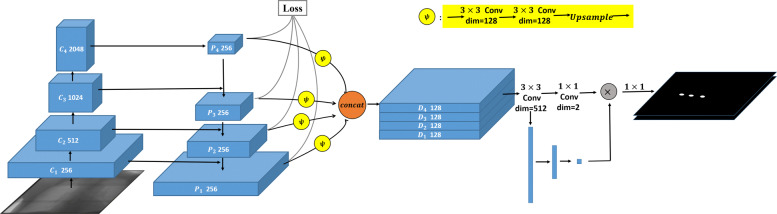
The architecture of LandmarkNet

The architecture of our proposed model, LandmarkNet, can be found in Fig. 1. In the following, we describe each component of the model in detail.

### The backbone

**Fig. 2:**
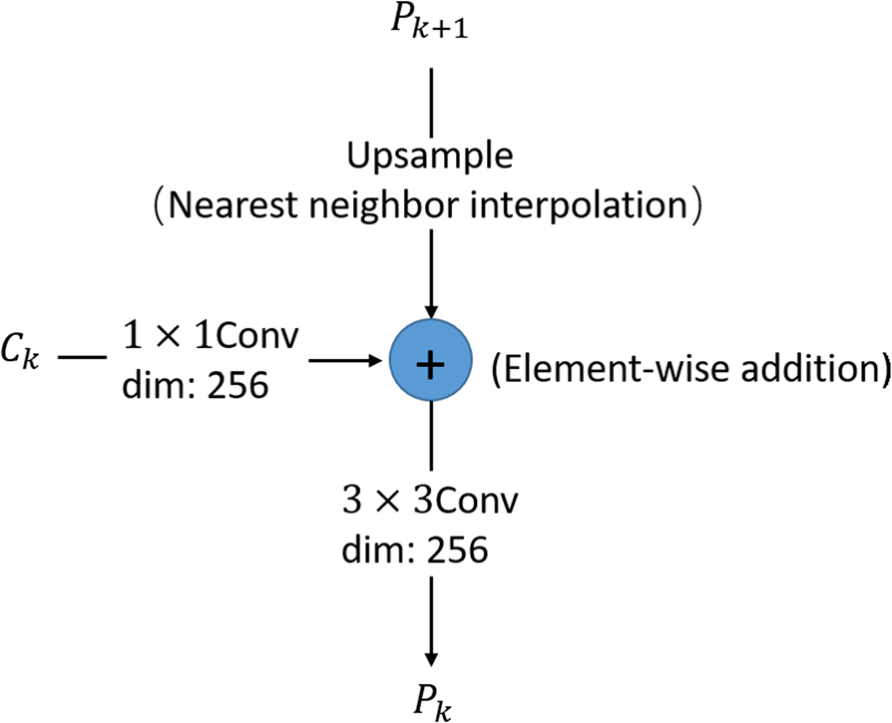
The details of lateral connection

The backbone of LandmarkNet uses the structure of Feature Pyramid Networks (FPN) [17] as a feature extractor. The bottom-up pathway (denoted by *C*) consists of four stages, the bottle neck block of ResNet is used in each stage. The network produces feature maps with the same size in every single stage and extracts features from the last residual blocks as {*C*_1_,*C*_2_,*C*_3_,*C*_4_} with strides of (4,8,16,32) pixels with respect to the input image. The hierarchical stage has such a property of reducing scale by twice and doubling the dimensions of the channel for feature maps. The architecture of bottom-up pathway is shown in Table 1. The top-down pathway (denoted by *P*) upsamples the resolution of feature maps {*P*_2_,*P*_3_,*P*_4_} with nearest neighbor upsampling by a factor of 2. The upsampled maps are reduced channel dimensions to 256 by a 1×1 convolutional layer, and then merged with corresponding bottom-up maps by element-wise addition, see Fig. 2. Furthermore, the bottom-up maps keep the same number of channels with the top-down maps via a 1×1 convolutional layer before adding. There are two output branches for each stage of top-down pathway, one for intermediate supervision, implemented by computing and back-propagating mean square error (MSE) loss between *P* feature maps and ground truth with corresponding resolutions. Another for lateral connections to estimate landmarks.

**Table 1:** The architecture of bottom-up pathway

Output layer	Output size	Block	Repeat
*C*_1_	120×120	1×1,64	3
		3×3,64	
		1×1,256	
*C*_2_	60×60	1×1,128	4
		3×3,128	
		1×1,512	
*C*_3_	30×30	1×1,256	6
		3×3,256	
		1×1,1024	
*C*_4_	15×15	1×1,512	3
		3×3,512	
		1×1,2048	

### The landmarks detection network

This part of the network has the same strategies as FPN, utilizing the hierarchical feature maps by lateral connection which undergoes two 3×3 convolutional layers to unified channel dimension to 128 and a nearest neighbor upsampling layer to resize feature maps to the same scale with input image, we denote this process by *φ*, and then concatenate them as a 512 dimension feature map (denoted by *D*). This architecture combines low-resolution, semantically strong features with high-resolution, semantically weak features by the hierarchical stage and lateral connections. Two subnets follow *D*, one of them produces *n* dimension feature map via a 3×3 convolutional layer and a 1×1 convolutional layer. The other generates a 1×*n* tensor via two consecutive full connected layers, where *n* is the number of types of landmarks. It is a mechanism similar to a gate in a recurrent neural network. A weight is generated for each feature channel by the parameter w, where the parameter w is learned to explicitly model the correlation between the feature channels. Finally, a reweight operation treats the weight of the output of Excitation as the importance of each feature channel after the feature selection, and then weights the previous feature by multiplication, completing recalibration of the original feature on the channel dimension. In our experiment, we choose *n* equal to 2, which represents different parts of the body, spinous process and tracheal bifurcation.

### Dataset

We obtained 8054 pairs of medical images of MV-DRs and KV-DRRs from several cancer hospitals in Zhejiang, and labeled the landmark annotations manually. we split the entire dataset into 65% training, 10% validation, and 25% test sets. This dataset includes images of skull, pelvis, leg bones and chest. Each patient radiographs from 0^∘^ and 90^∘^ respectively, and they are all 480×480 grayscale with three channels. The label is the position coordinates of the landmarks in the entire image.

### Training

We use Tensorflow to implement the model. We first resize images to 384×384 resolution as input and train on full images with batch size 18. The landmark annotations are converted into images with a different scale corresponding to different stage outputs of top-down pathway for computing intermediate loss. The MSE loss is applied to compare the predicted heatmap to a ground-truth heatmap consisting of a 2D Gaussian (with a deviation of 3 pixels) centered on the landmark location, because it is easy to calculate and optimize. The Adam optimizer with initial learning rate 1e-4 are used for computing adaptive learning rates for each parameter, and we choose default values of 0.9 for *β*_1_, 0.999 for *β*_2_, and 1e-8 for *ε*. The network is trained for 4000 epochs.

## Results

**Fig. 3:**
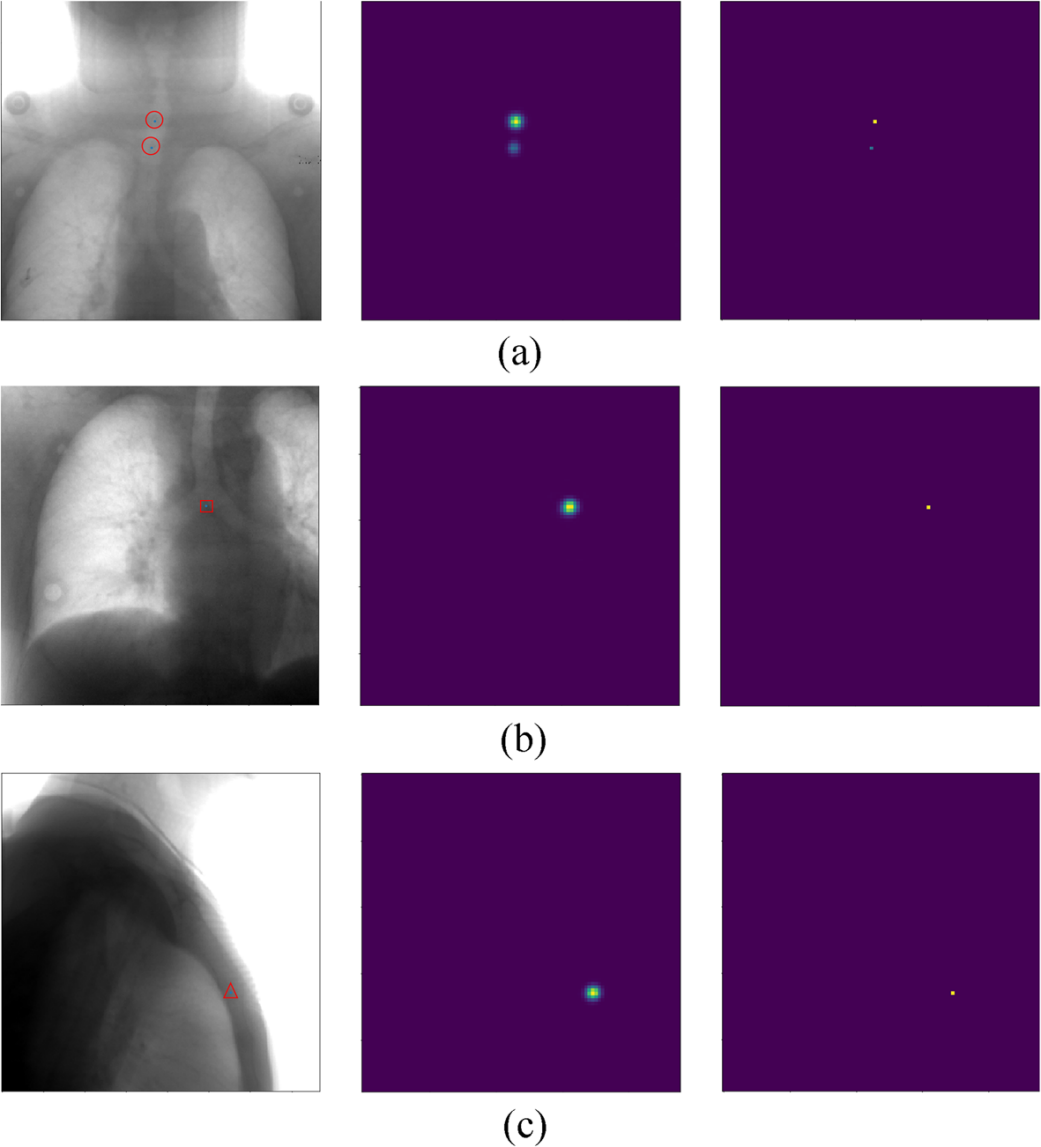
Example outputs produced by our network. **a**, **b**, **c** represent Spinous process, Tracheal bifurcation, Louis angle respectively. The images on the left are the inputs of our network. To show the points of interest, we manually marked them with different red geometric shapes and the landmark is located in the centroid of the geometry marked with blue dots. The middle images are the output heatmap of the network. On the right are the images processed by NMS

The example results produced by our network are shown in Fig. 3. Landmark’s location and quantity can be accurately predicted. Heatmap would predict the approximate location of the landmark with Gaussian distribution. Transform from a probabilistic map into a deterministic point. Slide a window through a given heatmap to find their maximum values, setting all the rest to zero. Each non-zero value of the resulting image represents the location of a landmark.

**Fig. 4:**
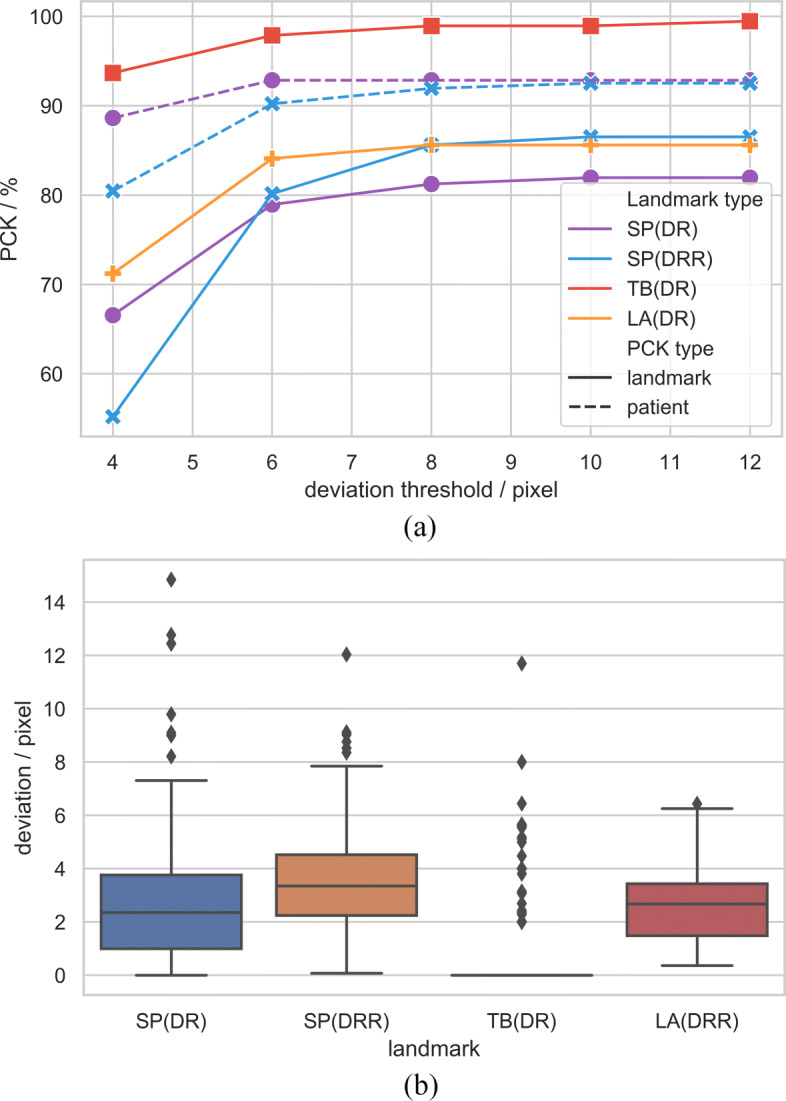
Result analysis. The line plot **a** shows the PCK within deviation threshold of (4, 6, 8, 10, 12) pixel. When the threshold is set to greater than 6, the accuracy will increase slowly and keep at a high level. The accuracy of single landmark estimation is generally higher than that of multiple landmarks. The box plot **b** shows the deviation for estimating different landmarks in images produced in different way. Our model works especially well when estimating tracheal bifurcation, and basically maintains a 0 deviation estimate. The performance on MV-DRs and KV-DRRs at the same landmark is similar, but there are less large deviations on KV-DRRs

Evaluation is done using the standard PCK metric which reports the percentage of detections that fall within 8 pixels distance of the ground truth in a 384×384 image. Results can be seen in Table 2 and Fig. 4. The landmark PCK represents the correct proportion of all landmarks in the output compared to the ground truth. When calculating patient PCK, as long as one of the landmarks is within the error range, we hold this result is accurate. For landmarks such as tracheal bifurcation and Louis angle, there is only one annotation in a single image, but there are usually three for spinous processes. When only part of the landmarks in a single image is accurately estimated, it would cause patient PCK is greater than landmark PCK. It is acceptable and helpful for registration as long as one landmark is accurate.

**Table 2:** Accuracy of landmark prediction within 8 pixel

	PCK (landmark)	PCK (patient)	Mean deviation/ pixel
Spinous process (MV-DRs)	81.24%	92.86%	2.38
Spinous process (KV-DRRs)	85.61%	91.95%	3.42
Tracheal bifurcation (MV-DRs)	98.95%	98.95%	0.98
Louis angle (KV-DRRs)	85.61%	85.61%	2.64

We trained other models, like DSNTr [18], Deeplabv3 [19], PRMs [20] on our own dataset. To adapt to our dataset, the last layer of these models are appropriately modified. The training is performed according to the parameters and methods proposed in the original paper. Evaluation only for trachea bifurcation, and the results shown in Table 3. Our model has higher accuracy. Other methods are more suitable for the detection of human feature points, and our method can achieve better performance for medical images.

**Table 3:** Comparison with the state-of-the-art methods

Method	PCK(%)	Mean deviation (pixel)
DSNTr [18]	97.01	2.99
Deeplabv3 [19]	97.74	2.11
PRMs [20]	98.13	1.45
Ours	98.95	0.98

## Discussion

From the previous section, we provide a model to automatically estimate keypoints in digital radiographs for registration. It is an indispensable and vital process in IGRT that comparing and aligning the in-room images taken before the treatment to the reference computed tomography (CT) scans taken during the planning phase. For images that are inaccurate in detail and not obvious, the previous work is the way to generate or reconstruct a new image from original unclear one, like [2], in which a complex model with a large number of parameters is proposed to predicted KV-DRRs from MV-DRs, then mutual information is used for calculating pairwise similarity to register the real KV-DRRs with predicted KV-DRRs. The multimodal registration is challenging for either clinicians or automatic algorithms, because not all pixels are helpful for registration. The surgeon also uses several characteristic points of the human body as the basis for registration. Our work is to process multimodal images by filtering out redundant information and only keeping key points, then register only with keypoints.

Similar key point detection has been applied in medical images, such as pulmonary nodules [3]. The detection of pulmonary nodules uses the method of object detection, which is a position estimation of a specific shape. Our model is to estimate the location of the key points of multiple features, equivalent to multi-category and multi-target detection.

There are several limitations to the current study. Firstly, we only select three landmarks to train and test. Only one landmark is estimated, in other words, no two kinds of landmarks are simultaneously estimated on one image. We need to find more kinds of landmarks with the help of doctors. Slightly increasing the number of key points helps to increase the reliability of registration and clinical application. The second limitation is that the accuracy of the estimate is not sufficient to be fully dependent and can only be used as a reference. We did not optimize for poorly estimated samples. We found some hard positive samples in the experimental results, but We have not conducted hard mining online or offline. This is a future question needing further research.

## Conclusions

This paper proposed a novel network, denoted as LandmarkNet, to handle the landmark estimation of blurred medical image problem. We design a novel network structure which uses lateral connections repeatedly. The experiments demonstrate that our contributions lead LandmarkNet to the state-of-the-art performance on the MV-DRs images, especially for tracheal bifurcation estimation.

## Data Availability

The datasets used and/or analysed during the current study are available from the corresponding author on reasonable request.

## Abbreviations

MV-DRs: Digital radiography generated by mega-level X-rays
KV-DRRs: Digitally reconstructed radiography re-projected from computed tomography of kilovolt X-ray
FPN: Feature Pyramid Network
NMS: Non-maximum suppression
PCK: Percentage of correct keywords
IGRT: Image-guided radiotherapy
MSE: Mean square error
CT: Computed tomography
SP: Spinous process
TB: Tracheal bifurcation
LA: Louis angle
